# Correlative atom probe tomography and scanning transmission electron microscopy reveal growth sequence of LPSO phase in Mg alloy containing Al and Gd

**DOI:** 10.1038/s41598-021-82705-3

**Published:** 2021-02-04

**Authors:** Koji Inoue, Kenta Yoshida, Yasuyoshi Nagai, Kyosuke Kishida, Haruyuki Inui

**Affiliations:** 1 Institute for Materials Research, Tohoku University, Oarai, Ibaraki 311-1313 Japan; 2 Department of Materials Science and Engineering, Kyoto University, Yoshida-Honmachi, Sakyo-ku, Kyoto, 606-8501 Japan; 3 Center for Elements Strategy Initiative for Structural Materials (ESISM), Kyoto University, Sakyo-ku, Kyoto, 606-8501 Japan

**Keywords:** Materials science, Physics

## Abstract

Atom probe tomography (APT) and transmission electron microscopy (TEM)/scanning transmission electron microscopy (STEM) have been used correlatively to explore atomic-scale local structure and chemistry of the exactly same area in the vicinity of growth front of a long-period stacking ordered (LPSO) phase in a ternary Mg–Al–Gd alloy. It is proved for the first time that enrichment of Gd atoms in four consecutive (0001) atomic layers precedes enrichment of Al atoms so that the formation of Al_6_Gd_8_ clusters occurs only after sufficient Al atoms to form Al_6_Gd_8_ clusters diffuse into the relevant portions. Lateral growth of the LPSO phase is found to occur by ‘ledge’ mechanism with the growth habit plane either {1$\overline{1}$00} or {11$\overline{2}$0} planes. The motion of ledges that give rise to lateral growth of the LPSO phase is considered to be controlled by diffusion of Al atoms.

## Introduction

Mg–M–RE (M: metal, RE: rare-earth elements) alloys such as those in Mg–Zn–Y and Mg–Al–Gd systems have received considerable attention as a new class of structural materials for many possible engineering applications due to their low weight, high strength and good ductility^1–3^. In these Mg–M–RE alloys, unique platelet precipitates so-called ‘synchronized’ long-period stacking ordered (LPSO) phases are known to form^4^. The synchronized LPSO phase consists of hexagonally-arranged close-packed atomic layers stacked along their plane normal ([0001] in the hcp (hexagonal closed packed) notation), in which M and RE atoms are enriched to form M_6_RE_8_ atomic clusters with the L1_2_-type atomic arrangement based on the fcc (face-centered cubic) stacking in four consecutive atomic layers that are bounded by a few Mg layers with the hcp stacking^4–12^. Here, it should be mentioned that since the LPSO phase is known to form in the Mg matrix so that their close-packed directions and planes are parallel to each other, Miller indices to express directions and planes for the LPSO phase are referred to as those of the matrix phase of Mg with the hcp structure unless otherwise stated. M and RE enriched layers and a few Mg layers constitute a block layer that corresponds to a structural unit, depending on whose stacking various polytypes are generated. Chemical modulation (enrichment of M and RE atoms) thus occurs synchronously with structural (stacking) modulation (either hcp or fcc stacking) so that the phase is called the ‘synchronized’ LPSO phase. Although the number of atomic layers enriched in M and RE atoms is fixed at four, various polytypes have been observed to form with different numbers of the bounding Mg layers. Those include 10*H*, 18*R*, 14*H*, and 24*R-*types in Ramsdell’s notation, which are formed with 1, 2, 3 and 4 Mg atomic layers, respectively^4^. For example, the stacking sequence of the 18*R*-type LPSO phase is described as A**BA/CB**CB**CB/AC**AC**AC/BA**B, where A, B and C denote three different stacking positions of close-packed atomic layers, bold letters indicate RE/M-enriched atomic layers and slash marks denote positions of stacking faults. The position of the stacking fault is always in the middle of the four consecutive atomic layers enriched with M and RE atoms because of the formation of M_6_RE_8_ atomic clusters of the L1_2_-type with the fcc stacking. The existence of the in-plane ordering of M and RE atoms in the four consecutive atomic layers together with pure Mg atomic layers indicates that the LPSO phase should be described with the concept of the order–disorder (OD) structure but not as a ‘LPSO’ phase in a strictly sense in crystallography^7–11^. Hereafter, the Mg–M–RE LPSO phases are designated as ‘LPSO/OD’ phases. LPSO/OD phases in Mg–M–RE alloys play a crucial role in providing high strength and good ductility to the low weight Mg–M–RE alloys. Therefore, the crystal structures and the deformation behaviors of these LPSO/OD phases have been the subjects of intensive studies in the last decade^4–16^. When compared with the extent of understanding for their crystal structures and the deformation behaviors, however, the formation process of these LPSO/OD phases have received less attention in spite of its technological importance, except for some works by scanning transmission electron microscopy (STEM)^9,17^ and by synchrotron X-ray diffraction^18,19^. We believe that more in-depth study with better three-dimensional (3D) spatial resolution for structure and chemistry is needed for better understanding the formation process of these LPSO/OD phases, such as how chemical modulation synchronizes with structural modulation.

STEM with angstrom spatial resolution, in particular Z(atomic number)-contrast imaging by high-angle annular dark-field (HAADF)-STEM has been provided various structural and chemical information of LPSO/OD phases on an atomic scale such as stacking sequences, enrichment of M/RE atoms and their in-plane ordering to form M_6_RE_8_ clusters^4–11^. However, this technique has its own limitation arising from the fact that the obtained atomic-resolution image is just a two-dimensional projection along a particular crystallographic direction so that 3D information lacks inevitably unless additional imaging along other directions is made. This means that even with Z-contrast imaging, chemical information obtained on atomic scale is only that averaged over each atomic column along the projection. On top of that, since the intensity of each atomic column is proportional to the square of Z averaged over atoms in the respective atomic column, differentiating atoms with close atomic numbers is usually very difficult to be made. Differentiating Mg (Z = 12) and Al (Z = 13) in Mg–Al–Gd ternary alloys is one of the typical cases. Atom probe tomography (APT), by which 3D elemental map can be reconstructed at (sub-) nanometer spatial resolution for a needle specimen^20–22^, is a unique method that possesses high analytical sensitivity independent of atomic number and high mass-resolving capability that can distinguish even isotopes. This means that APT can provide elemental information that is difficult to obtain simply by atomic-resolution Z-contrast STEM imaging. However, since the spatial resolution in APT analysis is material-dependent and is sometimes insufficient to capture crystallographic information on crystal and defect structures as TEM/STEM imaging can do. In view of the fact that the capabilities of TEM/STEM and APT analyses are complementary, their correlated application to the exactly same area of analysis with the use of an identical specimen can provide complete information on local structure and chemistry on an atomic scale^23,24^.

In the present study, we investigate variations of atomic structures and elemental distributions on an atomic scale occurring at the interface region between the LPSO/OD and Mg phases in a Mg–Al–Gd alloy by correlative atom prove tomography and TEM/STEM made in the exactly same regions, in order to better understand how chemical modulation synchronizes with structural modulation in the growth sequence of the ‘synchronized’ LPSO phase in Mg with better 3D spatial resolution.

## Results

### Correlated TEM and APT observations

**Figure 1:**
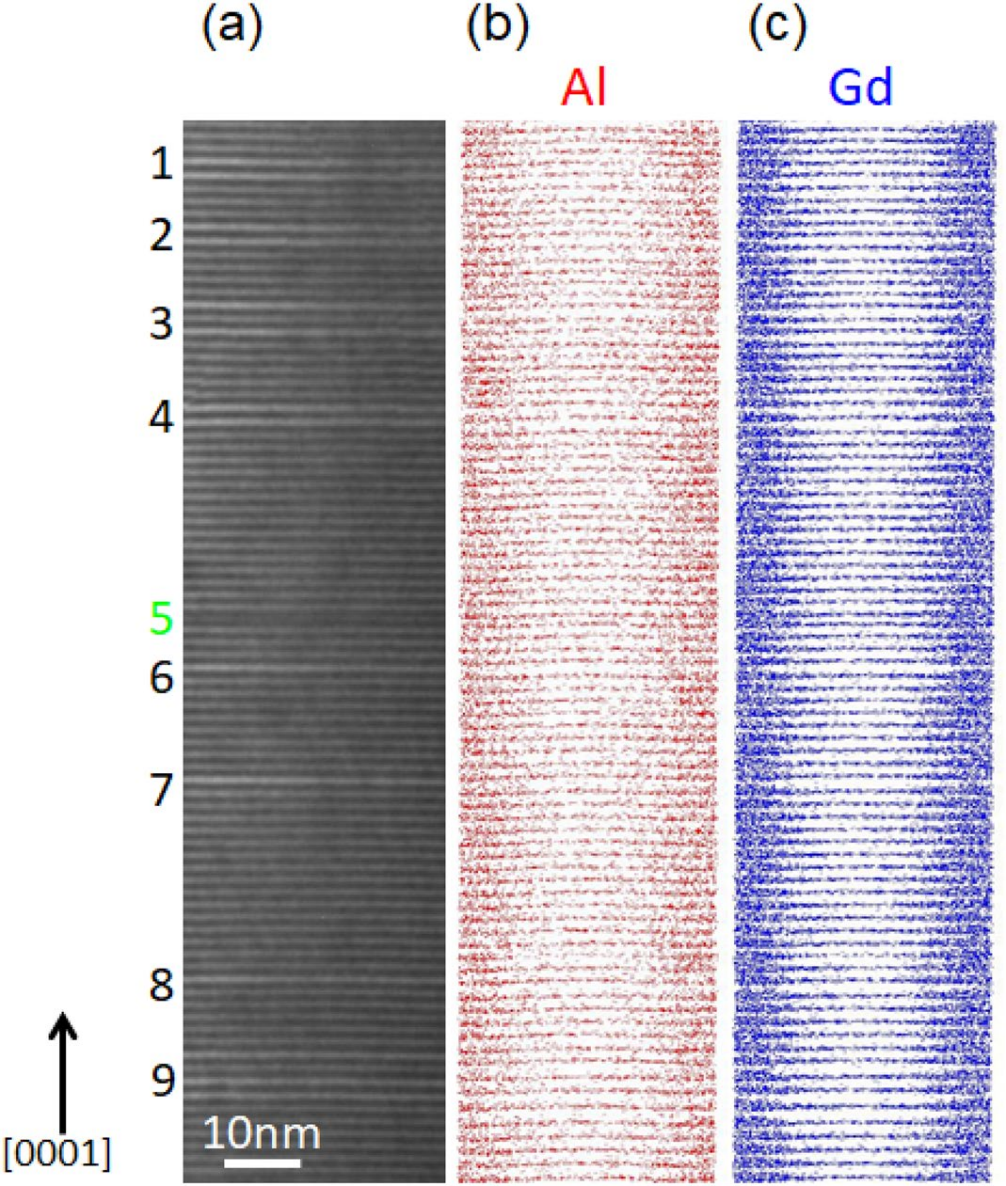
(**a**) TEM image (lattice fringe image by phase contrast). (**b**,**c**) 15-nm-thick sliced APT maps for Al and Gd in the same region of the TEM image. The vertical direction corresponds to the [0001] direction. This region includes local small part of 10*H* and 14*H*-type structure in the majority of 18*R*-type LPSO/OD structure. Number indicates the irregular spacing of enriched layers corresponding to the 10H-type structure for #5 or 14*H* structure for #1–4 and #6–9. Software: (**a**) Gatan Microscopy Suite (GMS), Ver. 2.30.542.0, https://www.gatan.com/products/tem-analysis/gatan-microscopy-suite-software, (**b**,**c**) Integrated Visualization and Analysis Software (IVAS), Ver. 3.6.14, https://www.cameca.com/service/software/ivas.

An interference-fringe TEM image formed with reflections of the [000*l*] systematic row and APT maps for Al and Gd in a 15-nm-thick slice selected from the whole maps obtained from an identical needle specimen of the LPSO/OD phase in the Mg–Al–Gd systems are shown in Fig. 1a–c, respectively. The vertical direction in Fig. 1 corresponds to the needle axis that is parallel to the [0001] direction of the LPSO/OD phase. The fringe spacing in the TEM image of Fig. 1a corresponds to a thickness of each of block layers consisting of Gd/Al-enriched atomic layers and Mg atomic layers. The fringe contrast occurs regularly at the interval of about 1.6 nm in most areas, conforming the occurrence of the polytype of the 18*R*-type that consists of Gd/Al-enriched four consecutive atomic layers and two Mg atomic layers. However, some irregularities are noted here and there, indicating the occurrence of other polytypes; those of the 14*H*- and 10*H*-types, in which three and one Mg atomic layers respectively constitute their block layers together with Gd/Al-enriched four consecutive atomic layers, are found at positions #1–4 and #6–9 and at a position #5, respectively, in Fig. 1a. Atomic layers enriched with Al and Gd are observed in the APT maps of Fig. 1b,c with some irregularities in their intervals. Surprisingly, the irregularities observed for the occurrence of atomic layers enriched with Al and Gd in the APT maps of Fig. 1b,c completely coincide with those observed for the fringe contrast in the TEM image of Fig. 1a. This clearly indicates that the irregularities for the occurrence of polytypes can be used as a marker to successfully make correlated TEM and APT analyses of the LPSO/OD phase in the Mg–Al–Gd system.

### Correlated atomic-resolution HAADF-STEM and APT observations

**Figure 2:**
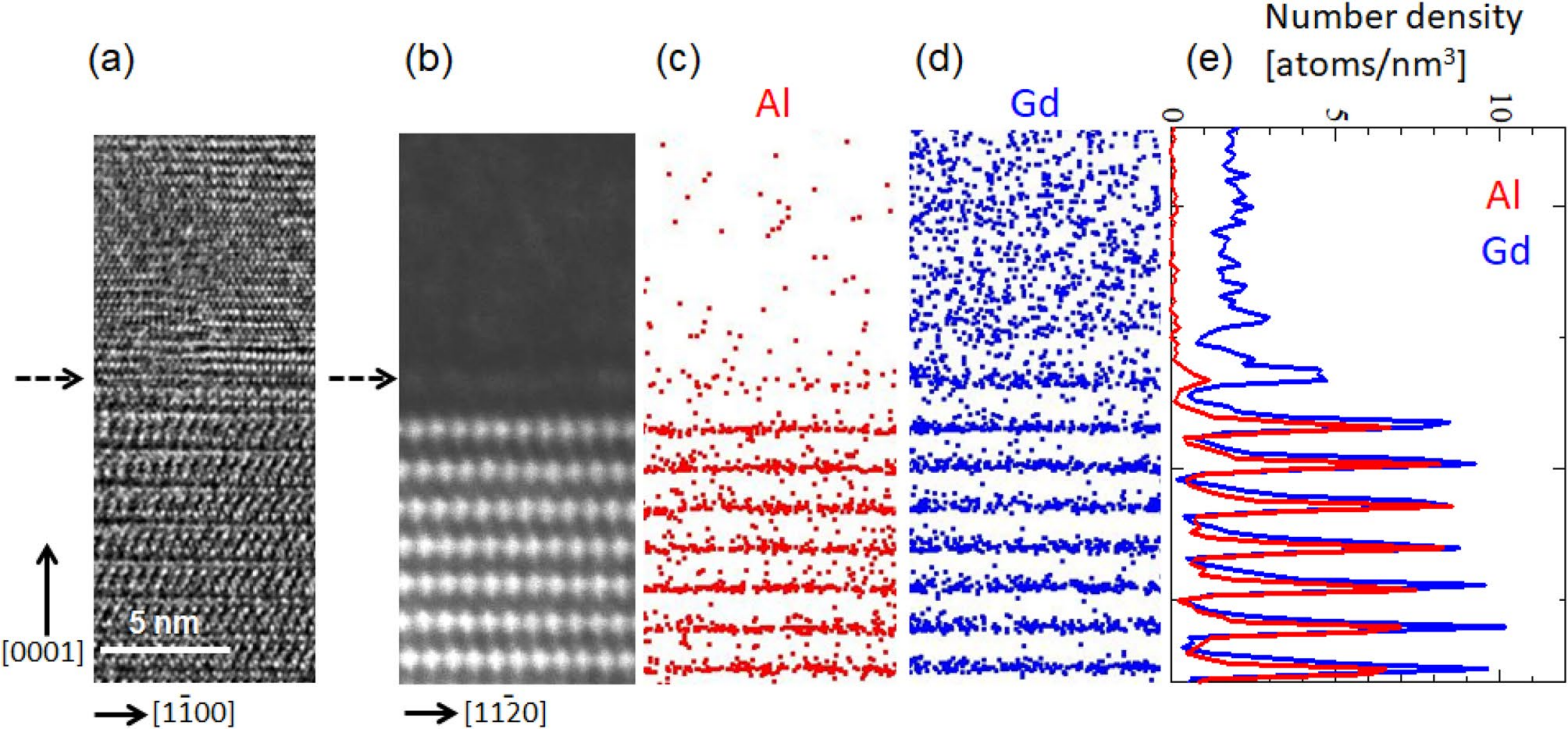
(**a**) High-resolution TEM image taken along the [11${2}( - )$0] direction. (**b**) HAADF-STEM image taken along the [1${1}( - )$00] direction. (**c**,**d**) 15-nm-thick sliced APT maps for Al and Gd in the same region as the TEM/STEM images. (**e**) Concentration profile along the [0001] direction around the interface region of the LPSO/OD and α-Mg phases. Software: (**a**,**b**) Gatan Microscopy Suite (GMS), Ver. 2.30.542.0, https://www.gatan.com/products/tem-analysis/gatan-microscopy-suite-software, (**c**,**d**) Integrated Visualization and Analysis Software (IVAS), Ver. 3.6.14, https://www.cameca.com/service/software/ivas, (**e**) Sma4 for windows, Ver. 1.58, http://hp.vector.co.jp/authors/VA002995/.

Figure 2a–d respectively show high-resolution TEM, STEM images and 15-nm-thick sliced APT maps for Al and Gd atoms taken from the exactly identical area including the interface between Mg and the LPSO/OD phase horizontally at the center of the images. Concentration profiles of Al and Gd atoms along the [0001] direction estimated from the APT maps is shown in Fig. 2e. The vertical direction in Fig. 2 corresponds to the needle axis that is parallel to the [0001] direction of the LPSO/OD phase. The incidence of the high-resolution TEM image is [11$\overline{2}$0], while it is [1$\overline{1}$00] for the HAADF-STEM image. Since the probability of successful APT analysis was found to decrease as the number of STEM observations increases (for example, by changing the incident beam directions) but not to decrease as the number of high-resolution TEM observations, only high-resolution TEM imaging with the [11$\overline{2}$0] incidence was made after the first STEM imaging with the [1$\overline{1}$00] incidence. In the Z-contrast HAADF-STEM image of Fig. 2b, Gd (Z = 64) atoms, which are far heavier than Mg (Z = 12) and Al (Z = 13), must be imaged brighter than any other atoms. In the HAADF-STEM image, an ordered arrangement of the bright spots with a so-called double-dagger pattern is observed in the region of the 18*R*-type LPSO/OD phase due to high degree of in-plane ordering of Al_6_Gd_8_ clusters with L1_2_-type atomic arrangement on the lattice points of a $2\sqrt 3 a_{{{\text{Mg}}}} \times 2\sqrt 3 a_{{{\text{Mg}}}}$ two-dimensional primitive hexagonal lattice in each structural block of the 18*R*-type LPSO/OD phase, as reported by Yokobayashi et al.^7^, where a_Mg_ refers to the magnitude of the a-axis of the hexagonal unit cell of Mg. The bright double-dagger markings of Al_6_Gd_8_ clusters in the LPSO/OD phase in the bottom half of the figure are observed to align regularly in their basal planes due to the in-plane ordering, forming bright marking rows parallel to (0001) and these bright rows appear regularly at the interval of the c-axis dimension of the structural block of the 18*R* type polytype. To be noted in the HAADF-STEM image of Fig. 2b, the occurrence of a faintly bright line parallel to (0001) planes at a distance of the c-axis dimension of the structural block of the 18*R* type from the top bright marking row of the LPSO/OD phase, as indicated by an arrow. In the high-resolution TEM image of Fig. 2a, the atomic layers in the region where a faintly bright line parallel to (0001) planes is observed in the HAADF-STEM image are observed to exhibit the hcp-type stacking. These indicate that in the thickening process of the LPSO/OD phase in Mg, enrichment of Gd atoms occurs on (0001) planes without disturbing the hcp stacking of Mg in the close vicinity of the interface between the LPSO/OD and Mg phases. This is consistent with previous observations by Kishida et al.^9^.

In the APT maps of Fig. 2c,d, the occurrence of enrichment of Al and Gd atoms on (0001) planes in a periodic manner is evident in the LPSO/OD phase in the bottom half of the figures. The occurrence of enrichment of Gd atoms on (0001) planes in the region where a faintly bright line parallel to (0001) planes is observed in the HAADF-STEM image is also evident from the correlative APT and STEM/TEM analysis. Of interest to notice is that in the region that exhibits a faintly bright line parallel to (0001), while Gd enrichment is clearly observed (Fig. 2c), Al enrichment is not as significant as Gd enrichment (Fig. 2d). Such information about Al distributions cannot be obtained by HAADF-STEM imaging because of the too close atomic numbers for Mg and Al atoms. This is further evidenced by the number density profiles of Al and Gd of Fig. 2e formed from the atom maps of Fig. 2c,d. In the LPSO/OD phase, the number density ratio of Al and Gd atoms in the (0001) row of Al_6_Gd_8_ clusters that exhibit bright double-dagger markings in the HAADF-STEM image is 1.39 ± 0.04 when averaged over 20 (0001) rows, which is a bit larger than that (1.33) expected from the formation of Al_6_Gd_8_ clusters. But, in the region that exhibits a faintly bright line parallel to (0001), not only the number density of Gd atoms but also the number density ratio of Al and Gd atoms is lower than those in the (0001) row of Al_6_Gd_8_ clusters in the LPSO/OD phase. All these indicate that in the thickening process of the LPSO/OD phase, enrichment of Gd atoms occurs first without disturbing the hcp stacking of Mg and that the formation of Al_6_Gd_8_ clusters accompanied by the stacking disturbance occurs only after sufficient Al atoms to form Al_6_Gd_8_ clusters together with Gd atoms reach to the relevant positions. The importance of diffusion of Al atoms in the formation of Al_6_Gd_8_ clusters was not noticed in the previous STEM study by Kishida et al.^9^ because of the incapability of STEM in differentiating Al from Mg atoms.

**Figure 3:**
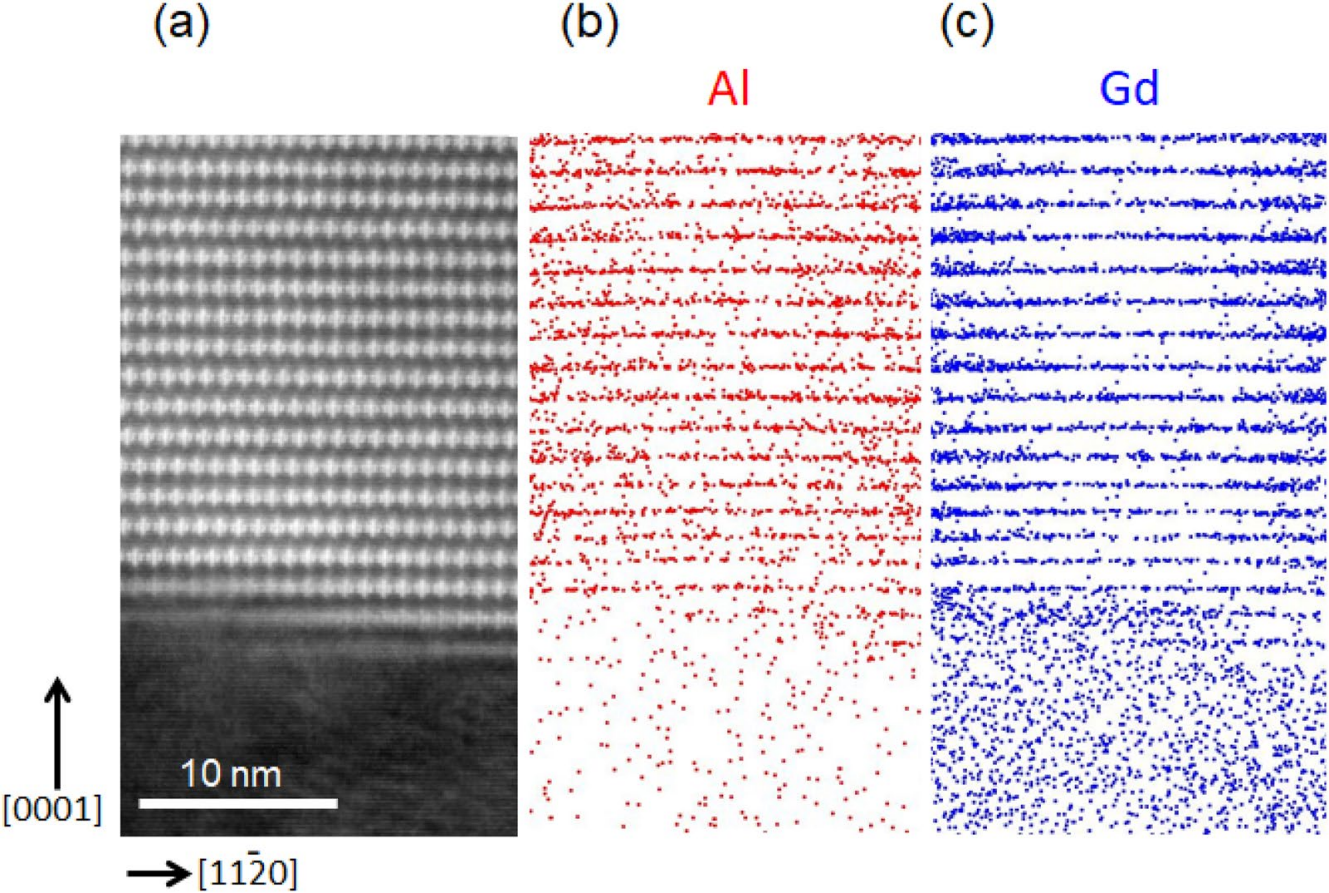
(**a**) HAADF-STEM image taken along the [1${1}( - )$00] direction. (**b**,**c**) 15-nm-thick sliced APT maps for Al and Gd in the same region as the STEM image around the interface region of the LPSO/OD and α-Mg phases. Software: (**a**) Gatan Microscopy Suite (GMS), Ver. 2.30.542.0, https://www.gatan.com/products/tem-analysis/gatan-microscopy-suite-software, (**b**,**c**) Integrated Visualization and Analysis Software (IVAS), Ver. 3.6.14, https://www.cameca.com/service/software/ivas.

Figure 3a–c respectively show HAADF-STEM image taken along the [1$\overline{1}$00] direction and 15-nm-thick sliced APT maps for Al and Gd in the vicinity of the interface between the LPSO/OD and Mg phases. (0001) Gd-enriched atomic layers in the transition region at the interface are observed to change in the HAADF-STEM contrast from a row of double-dagger patterns (right-hand side) to a slightly bright line (left-hand side) (Fig. 3a). In these (0001) Gd-enriched atomic layers in the transition region, while Gd enrichment is observed to extend across the areas where the HAADF-STEM contrast changes, Al enrichment is observed to be limited only in the layers where a row of double-dagger patterns is observed. This again confirms that the formation of Al_6_Gd_8_ clusters occurs only after Al atoms sufficient to form Al_6_Gd_8_ clusters reach to the relevant portions.

**Figure 4:**
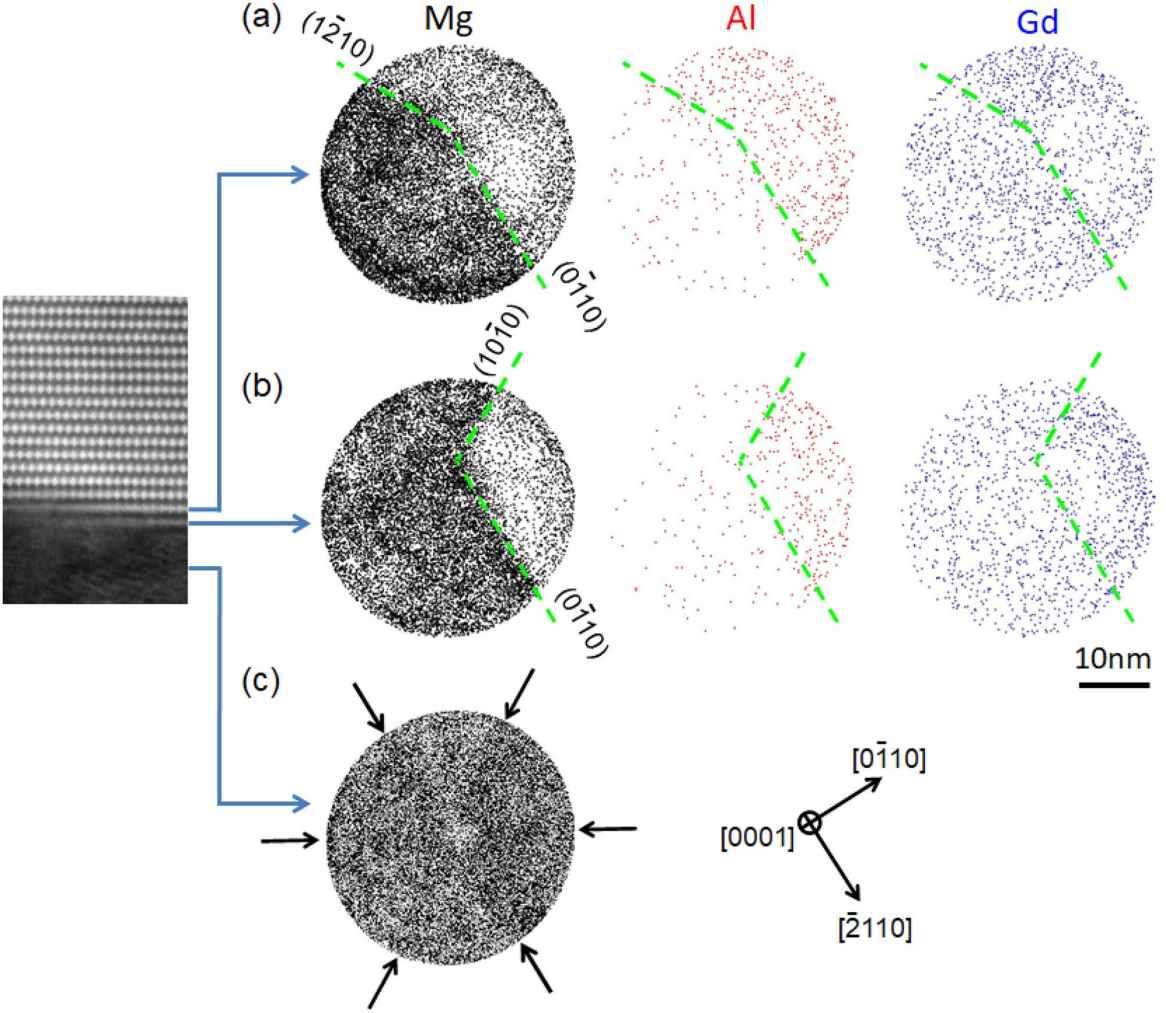
(**a**,**b**) Cross-sectional views of the APT maps at the enriched layers in the interface region of the LPSO/OD and α-Mg phases observed from the [0001] direction. The enriched layers correspond to those marked by the solid arrows in the HAADF-STEM image. (**c**) Cross-sectional view of the Mg map in the region of the α-Mg phase observed from the [0001] direction. The region of the α-Mg phase corresponds to that in the HAADF-STEM image marked by the solid arrow. The HAADF-STEM image is the same image as that in Fig. 3a. Software: (**a**–**c**) Integrated Visualization and Analysis Software (IVAS), Ver. 3.6.14, https://www.cameca.com/service/software/ivas.

Cross-sectional views of the APT maps are formed from the maps of Fig. 3b,c at three different sections shown in the HAADF-STEM image in Fig. 4 (the identical to Fig. 3a). Figure 4a,b are Mg, Al and Gd maps at the sections containing the transition regions with Gd-enriched layers, while Fig. 4c corresponds to Mg, Al and Gd maps at the Mg phase. Crystallographic directions on (0001) are determined from the pole pattern of the six-fold symmetry consisting of central < 0001 > pole and < 11$\overline{2}$0 > zone lines, as indicated in Fig. 4c ^25^. In Fig. 4a,b, the LPSO/OD phase regions are observed in the right-hand side as is evident from the higher and lower number densities of Al and Mg atoms, respectively. In the two sections of the transition region of Fig. 4a,b, the LPSO/OD phase region seems to have habit planes parallel to either {1$\overline{1}$00} or {11$\overline{2}$0} planes. This is consistent with the result from electron tomography experiment that lateral growth of the LPSO/OD phase in the Mg–Zn–Gd system occurs with habit planes parallel to either {1$\overline{1}$00} or {11$\overline{2}$0} planes with some preference of {11$\overline{2}$0}^26^. More work is definitely needed to unambiguously determine the growth habit plane (either {1$\overline{1}$00} or {11$\overline{2}$0} planes) for the LPSO/OD phase in the Mg–Al–Gd system.

## Discussion

We now discuss the growth sequence of the LPSO/OD phase in the Mg–Al–Gd alloy. The growth sequence seems to be consistent basically with that Kishida et al.^9^ proposed previously. They pointed out that (1) enrichment of Gd and Al atoms in four consecutive (0001) atomic layers keeping the hcp stacking, followed by (2) the formation of Al_6_Gd_8_ clusters with the L1_2_-type atomic arrangement in the four consecutive atomic layers introducing a stacking fault (fcc-type stacking) in the middle of the four consecutive atomic layers. In the present study, however, enrichment of Gd atoms in four consecutive (0001) atomic layers is observed to precede enrichment of Al atoms so that the formation of Al_6_Gd_8_ clusters occurs only after Al atoms sufficient to form Al_6_Gd_8_ clusters reach to the relevant positions. This means that the statement (1) is not wrong but that more strictly speaking, there is a sequential order for enrichment of Gd and Al atoms in the four consecutive (0001) atomic layers. This could be experimentally proved because of the capability of APT analysis to easily differentiate Al and Mg atoms. Although there is a sequential time lag for enrichment of Gd and Al atoms in the consecutive (0001) atomic layers, the introduction of stacking faults occurs only when Al_6_Gd_8_ clusters are formed after enrichment of Gd and Al atoms of full amounts completes in the relevant atomic layers. This means that chemical modulation (enrichment of Gd atoms) seems proceed by enrichment of Gd atoms without the disturbance of the stacking sequence, chemical modulation synchronizes with structural modulation by the stacking fault introduction due to Al enrichment to form Al_6_Gd_8_ clusters. Because the atomic radius of Gd (0.180 nm) is larger than that (0.150 nm) of Mg, the lattice misfit between the hcp Mg matrix and the four consecutive Gd enriched atomic layers is expected to arise, which should make the isolated occurrence of the layered enrichment of Gd unfavorable from the viewpoint of elastic strain energy. Therefore, the preceding layered enrichment of Gd preferentially at the growth front of the Mg–Al–Gd LPSO/OD phase as observed in the present alloy^9^ is considered to be assisted mainly by the chemical interactions between Gd atoms and the pre-existing plates of the Mg–Al–Gd LPSO/OD phase.

Kishida et al.^9^ proposed that the above processes (1) and (2) are followed by (3) the thickening by enrichment of Gd and Al atoms at a distance of two or three close-packed Mg atomic layers high from the pre-existing four consecutive layers so as to form another structural block. In the present study, this is confirmed to occur by ‘ledge’ mechanism with the growth habit plane either {1$\overline{1}$00} or {11$\overline{2}$0} planes. It has been revealed that a local concentration gradient of solute atoms, which is sufficient for the lateral growth of the precipitation phase by the ‘ledge’ mechanism, is developed in the vicinity of the ledge by diffusion^27^. In the case of the growth of the Mg–Al–Gd LPSO/OD phase, both Gd and Al atoms should diffuse into the growth front region. In view of the fact that Al_6_Gd_8_ clusters are not formed until a sufficient amount of Al atoms diffuses into the relevant position, the motion of ledges that give rise to the lateral growth of the LPSO/OD phase in Mg is considered to be controlled by diffusion of Al atoms. This should be reasonable because the concentration of Al in hcp Mg adjacent to the Mg–Al–Gd LPSO/OD phase is considerably lower than that of Gd as shown in Fig. 2e, even though the diffusion coefficient of Gd in Mg is about one order of magnitude lower than that of Al^28^.

They also proposed that (4) the in-plane ordering of Al_6_Gd_8_ clusters occurs so that they occupy regularly the lattice points of the two-dimensional $2\sqrt 3 a_{{{\text{Mg}}}} \times 2\sqrt 3 a_{{{\text{Mg}}}}$ primitive hexagonal lattice and the stacking of structural blocks (OD layers) changes to that energetically more favorable^9^. In the present study, the change in the HAADF-STEM contrast from a row of double-dagger patterns to a slightly bright line is proved to correlate well with the sudden change in enrichment of Al atoms (Fig. 3). This clearly indicates that once Al_6_Gd_8_ clusters are formed in the transition region, they tend to form in an ordered manner so that the in-plane ordering completes from the beginning. In the beginning of precipitation, however, while growth occurs keeping the in-plane ordering of Al_6_Gd_8_ clusters in each of structural blocks, the stacking of structural blocks is not yet that energetically favourable (as is evident from irregularities in the position of double dagger markings of Al_6_Gd_8_ clusters in structural blocks in the HAADF-STEM image of Fig. 3a). Precisely speaking, the change in the stacking of structural blocks occurs towards that energetically more favorable after the in-plane ordering of Al_6_Gd_8_ clusters in each of structural blocks in the statement of (4).

## Conclusions

The growth sequence of the LPSO/OD phase in a Mg–Al–Gd alloy have been investigated by correlative atom probe tomography and scanning transmission electron microscopy. The results obtained are summarized as follows.

1. Enrichment of Gd atoms in four consecutive (0001) atomic layers precedes enrichment of Al atoms so that the formation of Al_6_Gd_8_ clusters occurs only after sufficient Al atoms to form Al_6_Gd_8_ clusters diffuse into the relevant positions.
2. Lateral growth of the LPSO/OD phase occurs by ‘ledge’ mechanism with the growth habit plane parallel to either {1$\overline{1}$00} or {11$\overline{2}$0} planes. Since Al_6_Gd_8_ clusters are confirmed not to form until a sufficient amount of Al atoms diffuses into the relevant positions, the motion of ledges that give rise to the lateral growth of the LPSO/OD phase in Mg is considered to be controlled by diffusion of Al atoms.
3. Al_6_Gd_8_ tend to form in an ordered manner in the growth front so that the in-plane ordering completes from the beginning. The change in the stacking of structural blocks occurs subsequently towards that energetically more favorable.

## Methods

An ingot of a Mg–Al–Gd ternary alloy with a nominal composition of Mg–3.5 at% Al–7.0 at% Gd was prepared by high-frequency induction-melting and then annealed at 525 °C for 64 h. The upper part of the ingot with an approximate average chemical compositions of Mg–1at.% Al–5at.%Gd was used for TEM/STEM and APT analysis. The annealed ingot was composed of three phases, namely Mg, Al_2_Gd and Mg–Al–Gd LPSO/OD phase. The volume fraction of the Mg–Al–Gd LPSO/OD phase was confirmed to increase by both the thickening of the pre-existing plates and the nucleation of new precipitates accompanied by the elimination of GdMg_5_ additionally existed in the as-cast ingot. The details of the microstructure evolution during the annealing at 525 °C are described in Ref.^9^. Samples for TEM/STEM and APT analysis were cut from the ingot and needle specimens for TEM/STEM and APT analysis were fabricated by focused ion beam (FIB) machining using a FIB-SEM dual-beam system (Helios nanoLab600i, FEI). Prior to FIB machining, Pt coating was made on the sample surface to prevent damage by Ga-ion bombardment during FIB machining. Needle specimens were sharpened by an annular milling technique^29^, finishing with a low ion energy (2 kV) beam. Needle specimens were fabricated so that the needle axis coincides with the stacking direction (i.e., [0001]) of the LPSO/OD phase within the sample, since the spatial resolution along the needle axis is higher than that along the lateral directions.

Prior to APT analysis, microstructures of needle specimens were examined by conventional TEM (JEM-2000FX: JEOL) and spherical corrected TEM/STEM (ARM 200F: JEOL) respectively with acceleration voltage of 200 kV. Atomic-resolution TEM/STEM images were recorded with the incidence along either [11$\overline{2}$0] or [1$\overline{1}$00] of the LPSO/OD phase. Then, Ar ion milling at an acceleration energy of a few hundred eV (Gentle Mill: Technoorg Linda) was performed to the needle specimens for removing contaminations that had occurred during TEM/STEM observations. APT analysis was performed with a local electrode atom probe (LEAP4000XHR, Cameca) in the laser-pulsing mode. A pulsed laser with a 355 nm wavelength was used to irradiate the needle specimen with a repetition rate of 100 kHz and a pulse energy of 50 pJ. The base temperature of the needle specimen during the measurement was 30 K. Three-dimensional atom maps were reconstructed so as to correlative with the results of TEM/STEM observations with a dedicated software (IVAS, Cameca).

## Data Availability

The datasets generated during and/or analyzed during the current study are available from the corresponding author on reasonable request.
